# Primary immunodeficiencies in Bulgaria - achievements and challenges of the PID National Expert Center

**DOI:** 10.3389/fimmu.2022.922752

**Published:** 2022-09-22

**Authors:** Elissaveta Naumova, Spaska Lesichkova, Veneta Milenova, Petya Yankova, Marianna Murdjeva, Snezhina Mihailova

**Affiliations:** ^1^ Clinic of Clinical Immunology with Stem Cell Bank, Expert Center for Rare Diseases-PID, University Hospital “Alexandrovska”, Sofia, Bulgaria; ^2^ Department of Clinical Immunology, Faculty of Medicine, Medical University, Sofia, Bulgaria; ^3^ Department of Microbiology and Immunology, Faculty of Pharmacy, Research Institute, Medical University, Plovdiv, Bulgaria

**Keywords:** Primary immunodeficiency, Bulgarian PID Registry, epidemiology, genetic analysis, phenotypic characteristics

## Abstract

Tremendous progress has been made in the recognition of primary immune deficiencies (PIDs) in Bulgaria since in 2005 we have joined the J Project Central-Eastern European collaborative program. Ten years later an Expert Centre (ExpC) for Rare Diseases - Primary Immune Deficiencies at the University Hospital “Alexandrovska”- Sofia was established. In May 2017 The National Register of Patients with Rare Diseases also became operational as a database containing clinical and genetic information for Bulgarian patients with PID. The transfer of data and information on Bulgarian PID patients to the European Primary Immunodeficiency Database, managed by the European Society for Primary Immunodeficiency (ESID) has started in 2020. The total number of registered patients now is 191 (100 men and 91 women), with more than half of them being children (106; 55.5%). Regular updating of the information in the register showed that 5.2% of patients are deceased and the majority (94.8%) is a subject to continuous monitoring as it has been reported for other European countries as well. With the establishment of the ExpC, the dynamics in the diagnosis and registration of patients with PID significantly intensified. For a period of 5 years (2016-2021) 101 patients were evaluated and registered in comparison with previous period - before ExpC establishment when only 89 patients were diagnosed. The most common pathology was humoral immune deficiency (85 patients; 44.5%). Ninety-six (50.3%) of the patients underwent genetic testing, and 66. 7% had genetically confirmed diagnosis. Three of the variants have not been reported in population databases. Following genetic investigation confirmation of the initial phenotypic diagnosis was achieved in 82.8% of cases and change in the diagnosis - in 17%. Sixty-two patients were on regular replacement or specific therapy, and the rest received symptomatic and supportive treatment. In summary, we present the first epidemiological report of PIDs in Bulgaria, based on the National PID register. Data on the clinical, phenotypic and genetic characteristics of PID patients provided important information about the nature of primary immunodeficiency diseases in our country.

## Introduction

Primary immune deficiencies (PIDs) are rare diseases. According to the latest update of the International Union of Immunological Societies (IUIS) classification (1), mutations in 430 genes cause 404 different phenotypes of immunological diseases, divided into 10 groups based on the type of immunological defect. Their prevalence among the world’s population varies widely, from 1.51 (Germany) to 20.27 (Kuwait) per 100,000 (2, 3). In addition, the distribution of different PIDs also varies among populations. A number of factors are responsible for these variations and geographical location and structure of the populations, as well as the percentage of consanguinity marriages are among them. However, an important factor is the level of recognition of these rare diseases, and their registration in national and international registries (4, 5).

Bulgaria is a relatively small country with a population of 6,916,548 according to the National Statistical Institute data from 12.2020. The predominant ethnic population are Bulgarians who do not generally consanguinity relationships. However, there are also regions with closed communities (4% Roma ethnicity and 2% Turkish ethnicity), where the incidence of some PIDs such as Ataxia telangiectasia (AT) is increased. The first published case of PID in Bulgaria was described as dysgammaglobulinemia in 1965, and in 1997 our team diagnosed the first case of common variable immune deficiency (CVID) (6). However, more systematic work on the identification and registration of PID patients began in 2005 when Bulgarian immunologists became part of the Central-Eastern European collaborative program called J Project. The Expert Centre (ExpC) for Rare Diseases - Primary Immune Deficiencies at the University Hospital “Alexandrovska”-Sofia has been officially designated in April 2016 by order of the Minister of Health. In May 2017 the National Register of Patients with Rare Diseases, established and maintained by the National Centre for Public Health and Analysis (NCPHA), also became operational. The registry contains clinical and genetic information of PID patients. In 2020 the information on PID patients has also been included in the existing European Primary Immunodeficiency Database, managed by the European Society for Immunodeficiencies (ESID).

In this article we report for the first time the epidemiology of PIDs in Bulgaria, based on the National PID Registry, and analyze the factors which could improve the diagnosis and management of PID patients and their families. In addition, data summarized here will contribute to the improvement of our knowledge on these rare diseases of the immune system.

## Material and methods

### Work organization of the national register of patients with PID rare diseases

The software platform of the register has been developed by NCPHA, in accordance with the European legislation regarding personal data protection.

The register contains anonymous, clinical, laboratory and genetic data for subjects with PID and serves for epidemiological analysis in order to improve the management and treatment of PID patients. The structure of the database includes the following mandatory information: demographic data, family history, clinical and laboratory information on PID patients, genetic test results, age of onset of the disease and age of diagnosis. Treatment details were not obligatory part of the portfolio at the time of first patient registration, but were subsequently required. PID variants were grouped according to the IUIS classification from 2019 (1).

Patients with suspected PID were referred to the National PID ExpC for diagnostic conformation. The health structures from which patients were initially referred for diagnostic clarification were mainly university hospitals from all over the country, and more recently the practices of general practitioners (GPs). Data of patients with confirmed diagnoses were included into the registry by authorized persons working in the PID ExpC. Written informed consent form was signed by all registered patients or their legal guardians. The informed consent forms have been approved by the Ethics commission of the University Hospital “Alexandrovska” Sofia (protocol number 100A/13th of May, 2020). The registry data was updated regularly.

### Characteristics of patients

Patients were diagnosed according to the diagnostic criteria of ESID and IUIS (1, 7). Patients with secondary immune deficiencies were excluded. Epidemiological analysis included all registered patients until December, 2021.

### Diagnostic algorithm and tests

The algorithm for the diagnosis of PID has been developed by the National PID expert group and included the following stages: characteristic symptoms for PID derived from the patient’s medical history, screening tests, data from the patient’s clinical examination and patient referral to PID centers for more specialized studies, including immune phenotyping, functional and genetic tests. Patient’s course from symptoms’ emergence through diagnosis and treatment was outlined in detail with the cooperation of GPs, pediatricians, internal disease specialists, neurologists est. Diagnostic tests included: complete blood count, differential count, flow cytometry immune phenotyping with a wide range of monoclonal antibodies, serum immunoglobulins and their subclasses, antibody response to vaccines (Diphtheria and Tetanus toxoid, Pneumococcal polysaccharide and Haemophilus influenzae type B), assessment of T lymphocyte function (Dynabeads Human T-Activator CD3/CD28 Assay and Phytohaemagglutinin Stimulation Assay). Testing for autoantibodies (antinuclear antibody screening extractable nuclear antigen panel, antibodies associated with organ-specific autoimmunity, autoimmune cytopenias, vasculitis, coagulopathies etc.), phagocytic activity (flow cytometry dihydrorhodamine test), complement hemolytic activity (Classical and Alternative pathway hemolytic assay - CH50, AH50) and specific components of the complement was performed as needed. Additionally, other functional tests were performed, such as STAT/JAK/MAPK pathways activity and cytokine profiling (ProcartaPlex multiplex cytokine panels, Invitrogen™). Genetic research has been performed using up-to-date techniques, including Sanger sequencing, Next-generation sequencing (NGS), Whole genome sequencing, Fluorescent *in situ* hybridization (FISH), according to standard protocols and in collaboration with INVITAE (Invitae Corp. San Francisco, California, U.S.), GRID (Genomics of Rare Immune Disorders, UK) and other PID centres within Jeffry Model Centres (JMC) Network and J Project.

### Statistical methods

The data analysis was performed using descriptive statistics and the SPSSv16.0 program. A pair t-test was used to assess the difference in the age of onset of symptoms, age of diagnosis, delay in diagnosis, and gender in the group of patients with PID. P-value ≤0.05 was considered statistical significant.

## Results

### Characteristics of PID patients in the national registry

**Figure 1:**
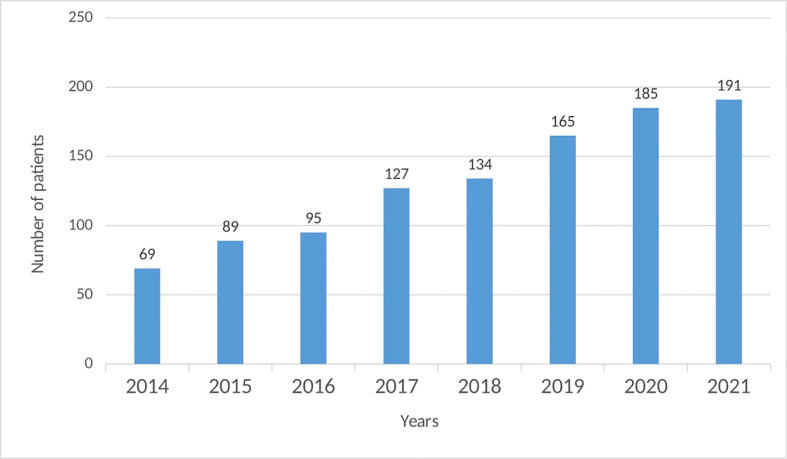
Distribution of the number of patients in the National PID Expert Center by years.

The Bulgarian National PID Registry includes 191 patients with PID, which represents a rate of 2.7 per 100,000. The last update is from December 2021. Until 2014 only 69 PID patients were diagnosed and registered in the National Registry (**Figure 1**). Since then, there has been an ascending tendency in the number of newly diagnosed patients, even more than doubled (177% increase).

The distribution of Bulgarian patients according to the updated IUIS classification from 2019 showed a predominance of antibody deficiencies (n=85, 44.5%) as those with CVID were 40 (20.9%), followed by selective IgA deficiency (n=22, 11.5%). Combined immune deficiencies (CID) with associated or syndromic features accounted for 17.3% (n=33), and individuals with auto-inflammatory diseases were 11% (n=21) of the patients. Seventeen patients (8.9%) remained without definitive diagnosis and represented a diagnostic challenge. Patients allocated to other PID categories make up less than 10% of all cases for the respective nosological group. Only 8 patients with hereditary angioedema (HAE) were included in the National Registry, classified to the group of complement deficiencies (n=11, 5.8%), as this entity is usually treated and monitored by specialists in allergic diseases. Diagnosed individuals with Immunodeficiency affecting cellular and humoral immunity were 10 (5.23%) and the same number of patients were registered with Congenital defects of phagocyte number, function or both. In the group of CID with associated or syndromic features, only 6 of 28 patients with AT from 4 ethnic families have been registered. The remaining 22 patients who have been clinically and genetically diagnosed with AT are currently pending to enter the registry so they were not included in this article.

**Figure 2:**
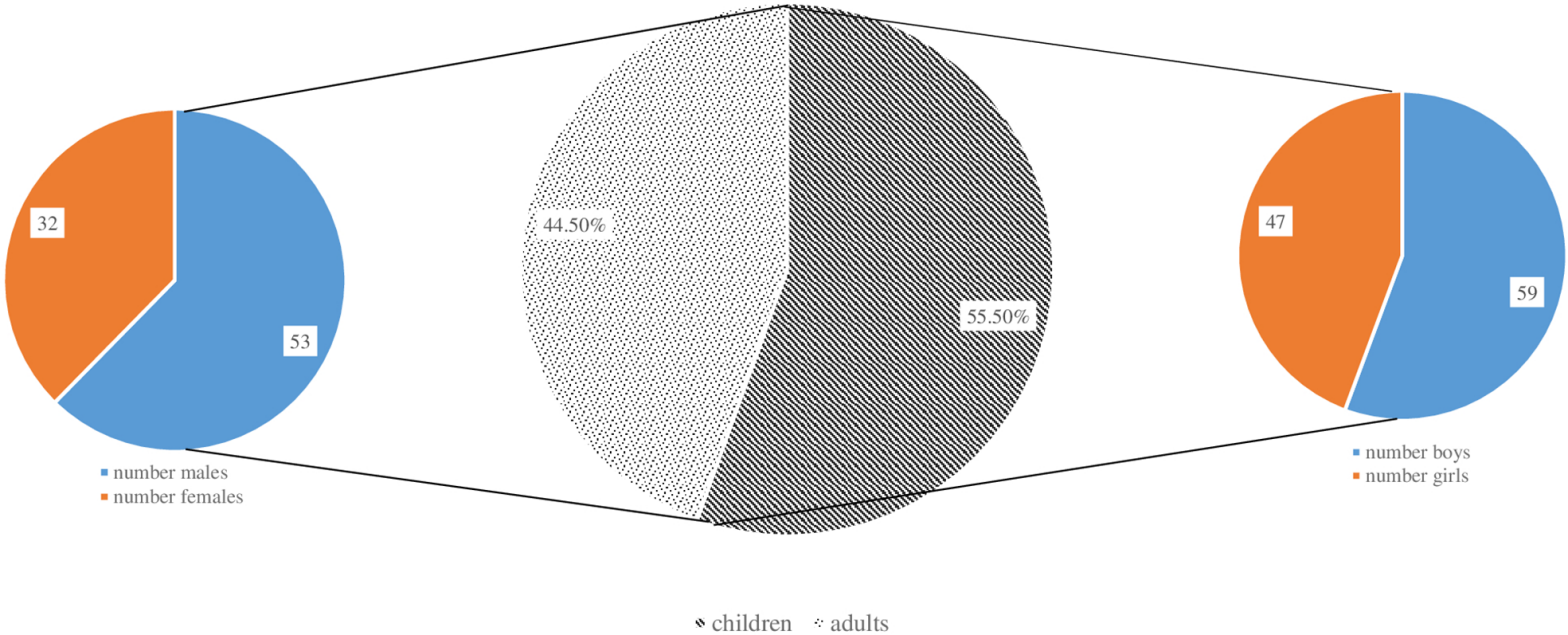
Distribution of patients in the National PID Expert Center by gender and age groups.

**Figure 2** shows the distribution of patients by gender and age groups. There is an almost equal ratio between males (n = 100) and females (n = 91). More than half of the patients are children (n = 106; 55.5%), 59 of whom are males and 47 - females. In the adult’s group males predominated as well. An extensive research of the patient’s medical history showed that in a large proportion of the adults (n = 40; 47%), the symptoms of primary immune deficiency dated back to childhood and adolescence. Ten (5.2%) of the registered patients were deceased and 181 (94.8%) were subject to long-term follow-up (**Table 1**). The distribution of patients by the age of onset of symptoms and the age of diagnosis showed that patients with severe combined immunodeficiencies (SCID) and CID with associated or syndromic features were diagnosed at 12 and 30 months (median age) after birth respectively, while the diagnosis of patients with antibody deficiencies was delayed with a median of 4 years (p <0.05). Greater delay in the diagnosis has been observed in patients with immune dysregulation diseases (median - 8 years) and in undefined PID patients (median - 5 years). The youngest diagnosed patients was a newborn with Bruton’s disease (genetic diagnosis was performed at birth due to positive family history of a brother with Bruton’s disease) and an infant with Hyper IgE syndrome due to clinical manifestations of eczematous pustular rash on the face and neck with fast dissemination, elevated markers of inflammation and microbiological data for methicillin-susceptible staphylococcus aureus, development of occipital and liver abscesses, pulmonary consolidation in both lungs and sepsis (genetic diagnosis at 4 months of age revealed the presence of STAT3 mutation). We also analyzed the delay in diagnosis according to gender. We found that in the group of patients with phagocytic defects the mean delay in diagnosis for females was 12.75 years, while for males was significantly lower (2.12 years; p=0,016) which is due to X-linked inheritance.

**Table 1:** Distribution of patients from the National PID Registry by main disease groups according to IUIS classification, sex, median age of onset and median age at diagnosis (diagnostic delay).

IUIS groups	Diagnosis	Patients(n)	Sex	Disease onset (median age;min-max)	Delay in diagnosis (median age; min-max)
1	Immunodeficiencies affected cellular and humoral immunity	10 (2ex)	M-5F-5	1m (0m -1y)	11m (0m-12y)
2	CID with associated or syndromic features	33 (3ex)	M-18F-15	2m (0m-10y)	1y (0m-46y)
3	Predominantly antibody deficiencies	85 (4ex)	М- 39F-46	5y (8m-54)	4y (0m-52y)
4	Diseases of immune dysregulation	4	М-2F-2	5y (1y-22y)	8y (4y-10y)
5	Congenital defects of phagocyte number or function	10 (1ex)	М-6F-4	0m (0m-31y)	1y (0m-22y)
6	Defects in intrinsic and innate immunity	0			
7	Auto-inflammatory disorders	21	М-14F-7	3y (3m-45y)	1y (0m-27y)
8	Complement deficiencies	11	М-7F-4	9y (7m-3y)	3y (5m-13y)
9	Bone marrow failure disorders	0			
10	Undefined	17	М-9F-8	3y (0m-43y)	5y (1y-62y)

M, male; F, female; ex.-exitus letalis.

### Epidemiology of PID in Bulgaria

**Figure 3:**
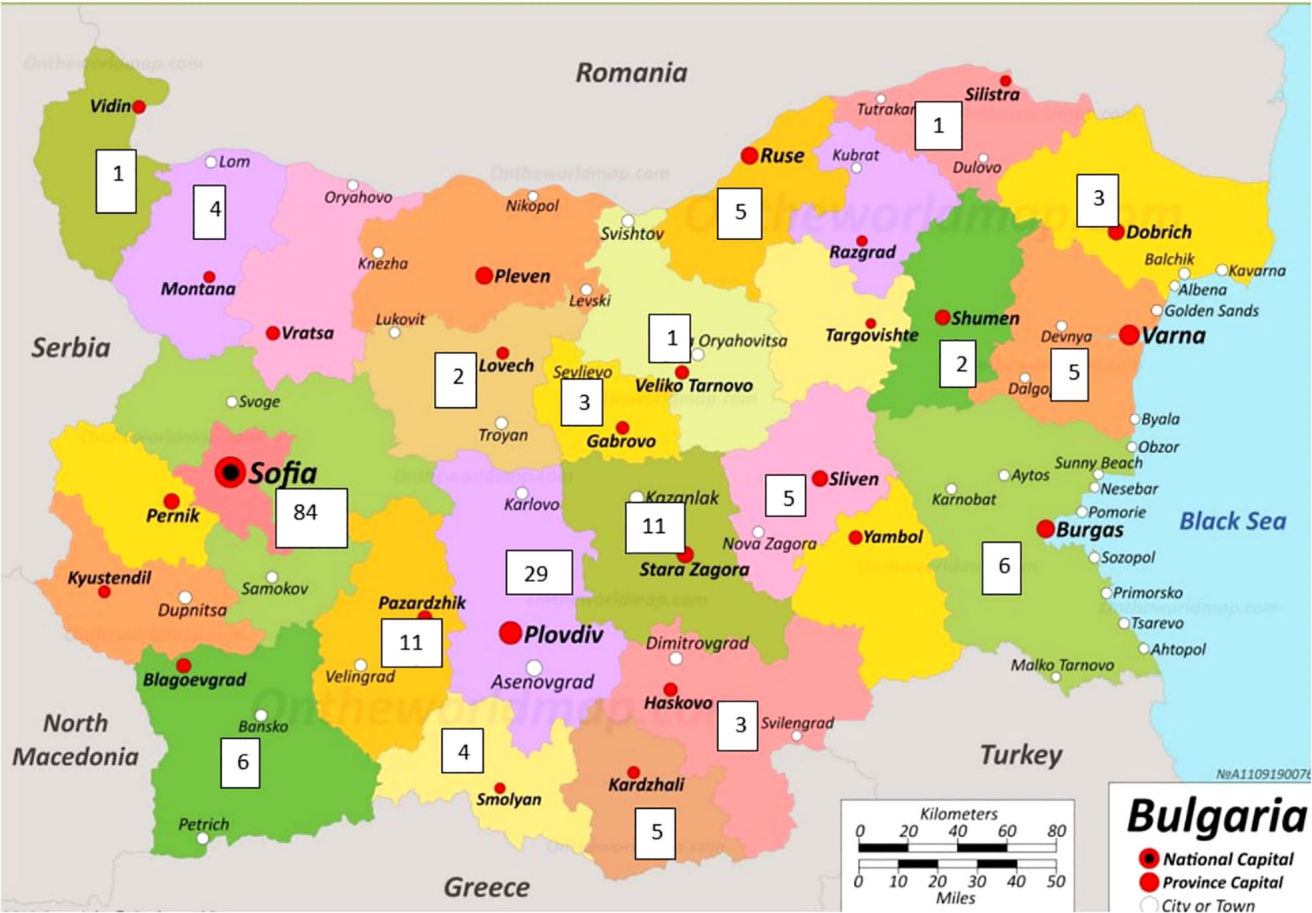
Geographical distribution of registered PID patients in Bulgaria (The map was adapted from https://ontheworldmap.com/bulgaria/).

The geographical distribution of PID on the territory of Bulgaria is based on patients included in the register (**Figure 3**). Patients were mainly concentrated in large cities such as Sofia with a population of 1,280,000 and Plovdiv with a population of 364 403 (North part of Bulgaria n=27/14.1%, South part of Bulgaria n=164/85.9%). The rest of the cases were distributed evenly in other districts of the country. It should be noted that in the municipality of Sarnitsa, Pazardzhik district, with a population of 3600 people, the majority of the population belongs to the so called Bulgarian Muslims ethnicity and the incidence of patients with AT (n = 28) is 0.7% of the population in the municipality, originating from 4 families.

### Genetic diagnosis of patients with PID

**Figure 4:**
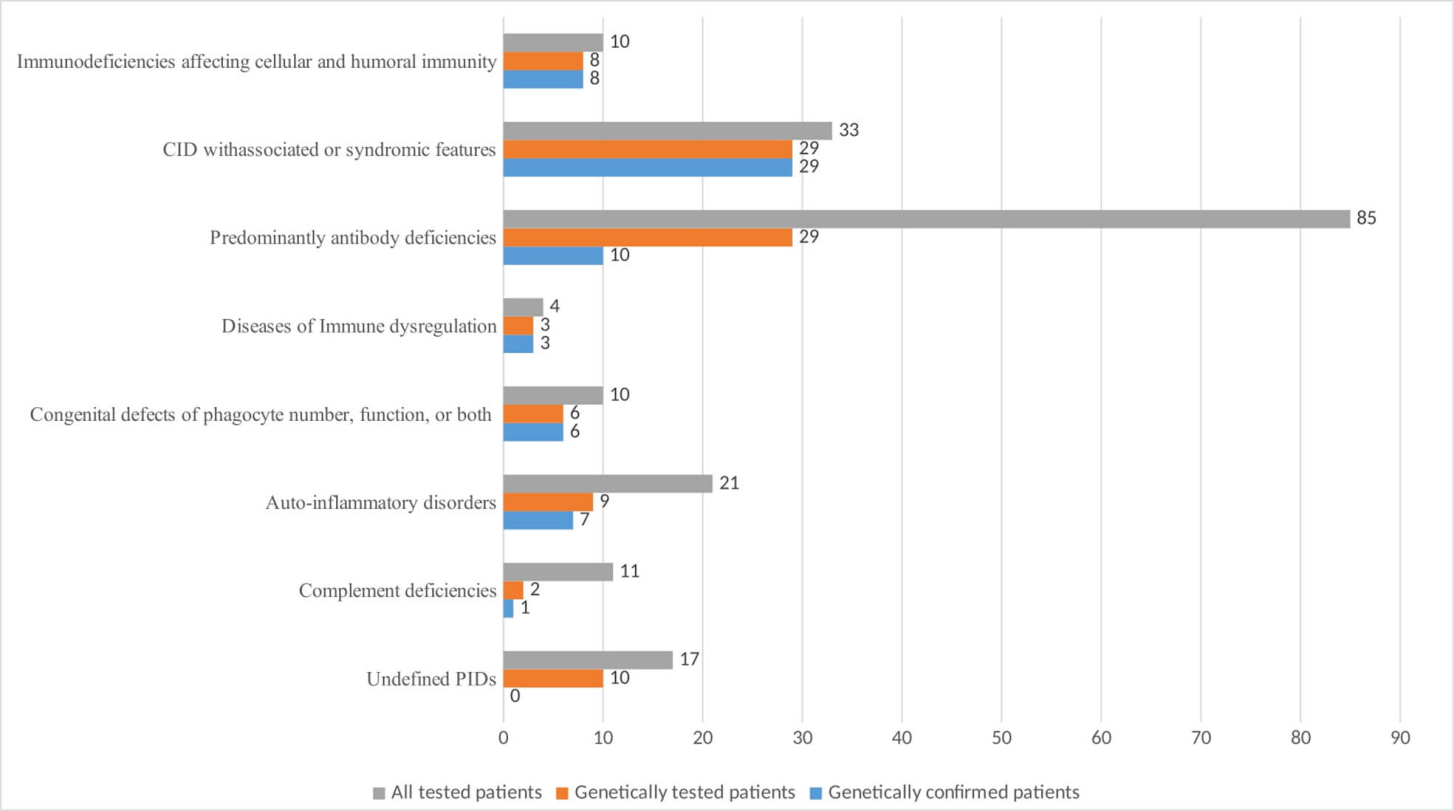
Number of PID patients by main classification groups with genetic testing and positive diagnostic confirmation.

Genetic testing was performed on 96 patients, representing 50.26% of the registered subjects (**Figure 4**). A mutation in one or more PID-related genes was found in 64 of them (66.7%), with the majority having autosomal recessive (AR) defects (50%). Autosomal dominant (AD) defects were established in 33.9% and X-linked defects in 16.1% of the cases. **Figure 4** demonstrates that the highest number of genetically tested patients relative to the total number of patients assigned to a PID category was in the group of CID with associated or syndromic features - 87.8%, followed by SCID - 80%, and the lowest number was in the group of patients with complement deficiencies (18%) and predominantly antibody deficiencies (34%). The analysis in different groups showed that pathogenic variants were entirely confirmed in all of the samples subject to testing if the patients had SCID, CID with associated or syndromic features, diseases of immune dysregulation and congenital defects of phagocyte number or function. In patients with antibody deficiency, the genetic diagnosis was positive in only 34.4% of those tested. Patients with undefined immune deficiency remained in this group due to the lack of molecular-phenotypic compliance, although over 58% of them were tested for pathogenic variants.

Detailed information referring pathogenic and likely pathogenic variants in selected patients included in the Bulgarian Registry with available exact molecular data is available as Supplementary material. Variants included missense mutations, duplications and deletion. Thanks to these data, a definitive diagnosis has been established. It is interesting to note that out of all patients diagnosed with CVID, only 4 had pathogenic variants related to CVID in a single gene TNFRSF13B in heterozygous state.

In 72 of all genetically tested patients with suspected PID, a targeted sequencing of a panel of genes associated with immune deficiencies (GRID program, INVITAE, TruSight One Sequencing Panels, Illumina) has been performed by next-generation sequencing technology, thus providing valuable information not only on the presence of pathogenic variants, but also on those of uncertain significance, some of which have certain clinical correlation.

This type of analysis gave us additional information on the carrier status of a total of 10 heterozygous pathogenic mutations associated with AR inherited diseases (**Table 2**). Three of the variants have not been reported in population databases (https://gnomad.broadinstitute.org/). The variant c.657_661del in the NBN gene was found by chance in three of the subjects who were not related to the patients with Nijmegen breakage syndrome included in the registry. This indicates a likely high frequency of carriage, requiring a larger number of tested individuals for accurate determination.

**Table 2:** List of pathogenic variants responsible for AR PIDs in patients tested with NGS technology in heterozygous stage.

GENE	VARIANT	STATE	Variant classification	Inheritance
TNFRSF13B	*c.311G>A (p.Cys104Tyr)*	heterozygous	Likely Pathogenic	AR
NBN	c.657_661del (p.Lys219Asnfs*16)	heterozygous	PATHOGENIC	AR
TCIRG1	c.1297C>T (p.Gln433*)	heterozygous	PATHOGENIC	AR
PTPRC*	c.308C>G (p.Ser103*)	heterozygous	PATHOGENIC	AR
TNFRSF13B	c.431C>G (p.Ser144*)	heterozygous	PATHOGENIC	AR
ICOS*	*Deletion (Entire coding sequence)*	heterozygous	PATHOGENIC	AR
LIPA	*c.894G>A (Silent)*	heterozygous	PATHOGENIC	AR
AK2*	c.597_599dup (p.Tyr200*)	heterozygous	PATHOGENIC	AR
TNFRSF13B	*c.260T>A (p.Ile87Asn)*	heterozygous	PATHOGENIC	AR
HPS3	*NM_032383.3:c.1101C>A*	heterozygous	PATHOGENIC	AR

*newly established variants.

In two of the patients with suspected PID, we have identified pathogenic variants which significance for phenotypic expression remains to be clarified. In a girl with T cell lymphopenia, deafness, and mild developmental delay, a pathogenic variant has been detected in the FTCD gene (NM_006657.2: FTCD c.990dupG), in homozygous state with TruSight One Sequencing Panel, Illumina. Mutations in this gene are responsible for the so-called glutamate formiminotransferase deficiency, a pathology with autosomal recessive inheritance, outside the PID group. The second case presented a girl with multiple clinical manifestations in which we have identified a pathogenic mutation in the KMT2D gene (NM_003482.3: c.5627A> C) in a heterozygous state associated with AD Kabuki syndrome 1.

The results from target-sequencing analysis of PID-related genes showed that a total of 265 variants were classified as Variants of Uncertain Significance. The variants included missense, nonsense, deletion, insertion and frame shift mutations. Thirty-nine of them have not been described yet in population databases. Fifty-six of the variants were in genes responsible for diseases characterized by AD or X-linked inheritance. This required multidisciplinary discussion, search for clinical-laboratory correlation,and follow-up of patients. Active monitoring of the status of identified variants from Uncertain to Benign or Pathogenic/Likely Pathogenic was required as well. Functional-diagnostic confirmation of the significance of these changes had been also considered.

We have also analyzed the correlation between phenotypic and genetic diagnoses. Confirmation of the phenotypic diagnosis was achieved in 82.8% of cases, and change in the diagnosis in 11 patients (17.2%). **Table 3** presents patients with non-compliance with the primary diagnosis based on phenotypic characteristics and genetic findings. Five of the patients remained in the same PID category, but with a different syndrome after genetic testing. Six patients were reclassified in another PID category. It is noteworthy that in patients with phenotypic hypogammaglobulinemia/CVID genetic testing often was the most useful and informative test for diagnostic clarification. Interestingly, in a child with a phenotypic characteristic of CVID and subsequent rapid development of non-Hodgkin’s lymphoma, the genetic diagnosis showed X-linked lymphoproliferative syndrome caused by a deletion in the SH2D1A gene. Following genetically-based re-classification, the therapeutic algorithm was re-evaluated in this particular case as well as in the majority of patients with diagnostic shift.

**Table 3:** Shift in the diagnostic categorization of patients after genetic testing, based on the identification of affected gene.

Diagnosis before genetic testing	Gene, in which the pathogenic variant was identified	Diagnosis after genetic testing	Treatment
Periodic Fever, Aphthous Stomatitis, Pharyngitis, Adenitis (PFAPA)	*MVK*	Mevalonate kinase deficiency (Hyper IgD syndrome)	Etanercept
Periodic Fever, Aphthous Stomatitis, Pharyngitis, Adenitis (PFAPA)	*MEFV*	Familial Mediterranean Fever (FMF)	Colchicine
Combined immunodeficiency	*RAG1*	Leaky SCID caused by hypomorphic mutation	HSCT
Nijmegen breakage syndrome	*NHEJ1*	SCID (T-B-NK+) Cernunnos	IRT
SCID-Omenn syndrome	*IL2RG*	X-linked SCID	IRT, Corticosteroids, Rituximab, Chemotherapy, HSCT
Chronic granulomatous disease (CGD)-female carrier	*MPO*	MPO deficiency	Antibiotics
Hypogammaglobulinemia	*CD40L*	Hyper IgM syndrome (HIGM)	IRT
Auto-inflammatory disorders	*MASP2*	MASP2 deficiencies	Antibiotics, immune modulators
Chediak-Higashi syndrome	*TBX1*	22q11.2 deletion syndrome	Symptomatic therapy
Common variable immunodeficiency (CVID)	*SH2D1A*	X-linked lymphoproliferative syndrome	IRT, Chemotherapy, Rituximab
Common variable immunodeficiency (CVID)	*CTLA-4*	CTLA-4 haploinsufficiency	IRT, Corticosteroids

IRT, Immunoglobulin Replacement Therapy.

### Therapy in PID patients

PIDs are complex diseases with multiple clinical manifestations, which determine the need for a comprehensive approach towards their treatment. Because antibody deficiency was the most commonly diagnosed cause of PIDs, regular immunoglobulin replacement therapy (IRT) was the primary and most available therapy. In our country the treatment for PID patients who need IRT or C1 esterase inhibitors is completely reimbursed by the National Health Insurance Fund (NHIF) since 2013. Fifty-four (28%) of our patients received Intravenous immunoglobulin/Subcutaneous Immunoglobulin (IVIg/SCIg), and in the last 5 years 91% of them received SCIg. One patient with CGD was treated with interferon gamma. Three patients received biological therapy for complications and two were treated with immunosuppressive therapy. Four patients were transplanted (SCID-2 and LAD-2) with good results, and in two others (Leaky SCID and Congenital neutropenia) the transplantation has been planned and forthcoming. The remaining patients were treated with long-term antimicrobial prophylaxis or anti-inflammatory therapy.

## Discussion

More than 400 million individuals worldwide suffer from a rare disease, and about half of them are children. As part of rare diseases, the incidence of PIDs in different populations varies from 1:2,000 to 1: 100,000. For European countries it ranges between 1.51 (Germany), 4.2 (Switzerland), Norway 5.3/100,000, United Kingdom at 5.90, and 8.0/100,000 (France) (2, 8–11)

According to the National Bulgarian PID Registry, the total incidence of PID in Bulgaria was 2.7/100,000, of which patients with predominantly antibody deficiencies were approximately 1.2/100,000, and SCID were 0.11/100,000 - a clear indicator of insufficient identification of various forms of immune deficiency. This is due to under-diagnosis of persons who have died before they were identified or cases with mild course that were missed. In reference to this, the establishment of specialized PID centres and registration of patients in registries at the national level is an important approach to more effective identification of PID cases (4). Other strategies are being implemented to better detection of PID patients, such as the creation of web-based networks to facilitate collaboration between GPs and PID experts (12). Here we present for the first time epidemiological, clinical and genetic data on PIDs in Bulgaria, based on the National PID register. All districts in the country were covered, which made this study a representative one for Bulgaria. It was not surprising that the majority of patients came from big cities with large population, such as Sofia and Plovdiv. However, it should be noted that access to specialized medical care, including clinical immunology service, is extremely easy due to the operation of specialized PID centres. On the other hand, there are remote areas in the country, such as the Rhodope Mountains, inhabited by the Bulgarian Muslims ethnicity - a religious minority, where 28 patients have been identified with AT, genetically verified and belonging to 4 families. Interestingly, despite the classical mutation in the ATM gene, these patients exhibited a phenotypic profile of long-term AT survivors. In addition, some PID entities were probably under-reported because they were mainly monitored in departments with specialties other than immunology, such as hemophagocytic lymphohistiocytosis, which is treated mainly by hematologists or auto-inflammatory diseases that are very common in rheumatologists’ practice. The data on the incidence of PID in Bulgaria should be interpreted carefully, due to the presence of prerequisites for under-diagnosis of PID patients. With the improvement of public awareness and collaboration between different medical specialists in the last 5 years, the detection of PID patients had improved significantly (almost twice). This was in line with the global trend in the PID community presented in the summary report from the JMC (12). Suspected patients were identified and referred to the PID ExpC in order to receive early and adequate diagnosis and treatment. The significant increase in the number of patients identified with immune deficiency was also due to the expansion of educational and awareness initiatives and the intensification of molecular diagnostics with current technologies. However, even more systematic steps are needed, including neonatal screening, prophylactic testing of immune competence at different stages of development of the children, adolescent and young adults, and improvement in the access to innovative treatment. We were already successful in using such approaches to identify patients with immune dysfunction. For example, our results from analysis of the post-vaccine immune response against protein antigens (tetanus and diphtheria) among a representative sample of the population showed (13) that individuals with insufficient titer of antibodies exhibited more frequent infectious and other symptoms and needed to be evaluated for the existence of primary or secondary immune disorders.

The distribution of PID diagnoses in Bulgaria was similar to that of the European Database (ESID), with a predominance of antibody deficiencies, followed by combined immune deficiencies with associated or syndromic features (14). Similar trend was also reported in the updated data for Russia (15) and Ukraine (16). Conversely, in Kuwait, the country with the highest incidence of PID (20.27/100,000), there was a predominance of severe forms of PID affecting cellular and humoral immunity followed by combined immune deficiencies with associated or syndromic features (3). According to the authors, this is due, on the one hand, to the improved patient identification due to the awareness of medical community, the well-organized referral of patients to clinical immunology service, and on the other hand - to the progress in the health system of the country. Similar factors were pointed at the base of the high incidence of PID in Iceland (18.8/100,000) - the country with the highest incidence of PID in Europe (17). Of course, in countries like Kuwait, the high incidence of consanguineous marriages must be taken into account, which was an explanation for the predominance of SCID (3).

On the other hand, data from the Consensus Middle East and North Africa Registry (18) showed that registered patients with inborn errors of immunity vary between 0.02 and 7.58 per 100,000 population. However, consanguinity in these areas are relatively high (60.5% of cases), and 27.3% of patients came from families with a confirmed previous family history of PID.

An important indicator is the delay between the onset of symptoms, diagnosis and initiation of treatment. Globally, it takes an average of 4.8 years to accurately diagnose a rare disease (12). Thirty percent of children with rare diseases will not survive until their 5th birthday, and thus serious steps are needed to improve this distressing statistic. In recent years, the delay in diagnosis has decreased significantly for the severe forms of PID in our country (under 12 months of age), thanks to the increased public awareness, significantly improved diagnostic capabilities, experience in PID and excellent collaboration between doctors from different medical specialties on national and international level. In comparison, the mean age of patients at baseline was 36 months and the mean delay in diagnosis was 41 months, according to the Middle East and North Africa Registry (18). It should be noted that the period from symptoms to diagnosis in patients with the most common clinically manifested immune deficiency – CVID, is the same to that in Germany (4 years) and shorter than in France and Sweden (6 years) (3, 8, 11).

At the same time, the diagnosis of the most severe form of immune deficiency, SCID, is extremely insufficient (0.11/100,000). This is a clear indicator of the need to implement a screening program for T- and B-cell deficiencies at a national level, for which there are already prerequisites in our country. A pilot study has been conducted to screen newborns with modern molecular technology in order to examine a dry blood spot taken at birth (Granted by Scientific Research Fund, Ministry of Education and Science). The data from the first stage of the study showed that 3 per 1,000 newborns were suspected of having T- and/or B-cell deficiency and were suitable for further clarification and follow-up (data not published). The results obtained on the prevalence of these diseases are a good basis for planning the necessary annual costs. Steps are to be taken for their inclusion in normative acts for mass genetic screening in the Republic of Bulgaria.

The entry of patient`s data from the Bulgarian Registry into the European database began in 2020. So far, 166 patients have been registered, which represents 86.9% of the patients in the National PID Registry. The process was slowed down by the fact that there were patients, mostly adults, who did not agree to consent to the registration of their personal data. Additionally, the necessary data and registration documents cannot be collected for some patients from remote areas of Bulgaria.

Our first analysis of Bulgarian patients with PID showed genetic diversity and a relatively high rate of confirmation of genetic diagnosis in 33.5% of all registered patients. The statistics was quite close to ESID Registry data, pointing genetic confirmation in approximately 36-43% of PID patients (French and German registries) (2, 11) and was significantly lower in comparison to the registered patients from the Russian population (49%) (15). The most common genes in which PID-related pathogenic variants were identified did not differ significantly from those reported for European populations. For example, 33% of the genetically tested patients diagnosed with CVID were carriers of a pathogenic mutation, data very similar to those reported by the German Registry (2). The highest percentage of genetically diagnosed patients had deletions in chromosome 22q11.2 - a total of 12 cases, followed by patients with a defect in the NBN gene and BTK gene - 5 (7.8 of the genetically proven). The observed tendncy for high frequency of heterozygous carriers of the so-called Slavic deletion in the NBN gene (c.657del5; rs587776650) - 4.16% of those tested, is not surprising for the Bulgarian population as this mutation is essentially characteristic for Slavic populations and therefore might be considered a Slavic founder mutation (18). High prevalence in the range of 0.5% to 1% of heterozygous carriers of c.657del5 was reported in population of Poland, Czech Republic/Slovakia, Ukraine and Germany (19, 20).

Despite the small number of detected and registered patients with SCID, the identified patients with defects in RAG and ADA prevailed over those with IL2R defect, a constellation different from the usual frequency of different forms of SCID. It is noteworthy that so far in our register there is no patient with a mutation in the WAS gene, given that the average incidence of Wiskott-Aldrich syndrome is between 1 and 10 cases per million boys. The aim of the diagnostic algorithm for the patients included in the registry was to use approaches based on NGS technologies for PID-related gene panels (75% of those tested were studied with this approach). This allowed us to detect and monitor patients with clinical and laboratory data corresponding to the so-called variants classified as “uncertain”, as well as follow-up the clinical manifestations in patients that are heterozygous carriers of pathogenic mutations responsible for AR diseases. The outstanding benefits of molecular diagnosis in patients were demonstrated with the change in the initial diagnosis in 11.4% of the tested patients, which also affected their therapy and prognosis. Patients with clinical and laboratory data of impaired immune response in whom the immune deficiency is classified as undefined are eligible for larger-scale genetic testing or phenocopies of PID should be suspected.

An extremely important issue is the treatment of PID. 95% of rare diseases lack FDA-approved treatment (12). With the introduction of IRT in the 1950s, the treatment of patients with predominantly antibody deficiency has been provided in a number of countries around the world. The NHIF in our country completely reimburses the treatment for PID patients who need IRT and C1 esterase inhibitor since 2013. Improving the access to immunoglobulin therapy is a persistent trend in all centres in Central and Eastern Europe. This is due to both the increase in the total number of patients and to a slight increase in the percentage of patients treated with immunoglobulins that is 19 to 23%, in the cumulative review of all centres participating in the Jeffrey Modell Foundation Central East Euorope network (21). The percentage of our patients treated with IVIg/SCIg was slightly higher (28%), and in the last 5 years the use of SCIg had significantly increased (more than 90% receive their therapy at home). This tendency is in line with the global shift towards personalized patient care, enabling treatment adjustment in order to ensure the best possible lifestyle. The number of patients with access to biological therapy and stem cell transplantation is also increasing. Unfortunately, in Bulgaria there is still no experience in transplanting children with PID, so up to date the cases had been transplanted abroad but funded from the NHIF. Treating patients with specific immune dysregulation due to a known genetic defect and treating the clinical manifestations of each individual patient is also a challenge. However, we are still experiencing difficulties in the application of biological and other modern methods of treatment due to lack of experience or lack of access or authorization, especially when “off-label” drug use is required. In this regard, the collaboration between the centres is of great importance for the benefit of patients.

The contribution of the two international projects that supported the work in the field of PID in our country needs to be outlined. In 2005, we joined a program for cooperation in Central and Eastern Europe in the field of physician education and clinical trials and aimed to improve the diagnosis and clinical care of patients with PID diseases, known as the J Project (22). The second program was developed by the Jeffrey Modell Foundation. In 2015 The Functional High Expertise Centre was established for training, development and improvement of diagnosis, treatment and care of patients with primary immunodeficiency diseases in Sofia, part of an international network of JMC (23). As a result, public awareness and collaboration between individual specialists such as pediatricians, rheumatologists and immunologists have significantly improved. The path from symptoms to diagnosis and treatment has emerged. In addition, in 2017 JMF developed a point system for assessing the risk of immune deficiency (12). This strategy reduces the uncertainties associated with the primary risks of immune deficiency, as we can test, identify and treat undiagnosed patients. It also allows for better consideration of regional differences and the distribution, age, gender, terms and conditions of access to qualified medical care and treatment. Last but not least, there are socio-economic benefits. On the other hand, IPOPI developed a PID Patient Life Index (24) to measure the state of implementation of the six PID principles (diagnosis, treatment, universal health coverage, specialist centers, national patient organizations and PID registries). It was emphasized that in countries without immunologists, patients with PID are at risk of being undiagnosed or misdiagnosed, leading in health implications or even death.

## Limitation of the study

This study has some limitations mainly related to the underdiagnosis of PID patients. Epidemiological and genetic analyzes were performed only on the basis of the patients diagnosed so far.

## Concluding remarks

The Bulgarian PID Registry provided for the first time epidemiological, clinical and genetic data that contributed to the understanding of the history and nature of PID in Bulgaria. Moreover, the establishment of Expert Centers had led to a systemic interest in PIDs and advances in their management in our country. The progressive increase in the number of newly registered cases underlined the improvement of PID awareness not only among the medical community but also among the public community. Continuous updating of the registry with new data and maintenance/updating of existing data ensured access to systematic and detailed information in a timely manner. This opportunity contributed to better understanding of PID and ensured improved diagnostic and treatment protocols for Bulgarian patients. The Expert Centre for Rare Diseases - PID in the University Hospital “Alexandrovska” - Sofia had a significant contribution in this process, providing Bulgarian patients with PID with equal access to health care facilities. This was possible due to great energy and enthusiasm of scientists and medical doctors, as well as the introduction of new diagnostic and therapeutic tools. In addition, we need to offer easy access to updated PID information regarding PID for doctors. The role of the patient organization “Bulgarian Association of People with Primary Immune Deficiencies” was also important, as it was related to advocacy, education and patient support.

We believe that our efforts will contribute to the development of a long-term strategy by the health authorities in order to provide the necessary resources to improve outcomes in prevention, treatment and management of PID patients in Bulgaria and ultimately improve patient care.

## Data availability statement

The data presented in the study are deposited in the ClinVar repository, submission numbers SCV002573411 – SCV002573436.

## Ethics statement

Written informed consent form was signed by all registered patients or their legal guardians. The informed consent forms have been approved by the Ethics commission of the University Hospital “Alexandrovska”, Sofia.

## Author contributions

EN conceived the original idea and wrote the manuscript with input from. EN, SM, SL, PY, and MM contributed to the diagnosis and clinical and immunological follow-up of the patients. VM collected and managed the data from the National PID Registry. MM contributed to the editing of the manuscript. All authors contributed to the article and approved the submitted version.

## Funding

This work was partly supported by ESID grant, 2019 and JMF Contract, 2015-2020.

## Acknowledgments

We thank all the teams from the JProject Centers and especially the Coordinator Laszlo Marodi, and the JMF CEE Network, particularly Vicki and Fred Modell. We express our gratitude to colleagues from health structures in the country, including university ones, for referring patients with suspected PID to National PID ExpC and inclusion in the national PID registry. We also thank patients and their families for their admirable approach to their rare diseases as well as the patient’s PID organization.

## Conflict of interest

The authors declare that the research was conducted in the absence of any commercial or financial relationships that could be construed as a potential conflict of interest.

## Publisher’s note

All claims expressed in this article are solely those of the authors and do not necessarily represent those of their affiliated organizations, or those of the publisher, the editors and the reviewers. Any product that may be evaluated in this article, or claim that may be made by its manufacturer, is not guaranteed or endorsed by the publisher.
