# Supplementary material for: Primary immunodeficiencies in Bulgaria - achievements and challenges of the PID National Expert Center
Source: Front Immunol. 2022 Sep 22;13:922752. doi: 10.3389/fimmu.2022.922752 (PMC9535737; doi:10.3389/fimmu.2022.922752)
Supplement: Supplementary file 1 [file DataSheet_1.pdf]

Molecular data of PID patients with known pathogenic or likely pathogenic genetic defect.

Gene	Mode of inheritance	Number of patients	Molecular defect	Type	Comment
**IMMUNODEFICIENCIES AFFECTING CELLULAR AND HUMORAL IMMUNITY**					
IL2RG	*X-linked*	1	*NM_000206.2:c.41T>C*	Likely pathogenic	This variant is not present in population databases (ExAC no frequency).
RAG1	AR	1	*NM_000448.3:c.1331C>T (p.Ala444Val)*	Pathogenic	
ADA	AR	1	*NM_000022.4:c.646G>A (p.Gly216Arg)*	Pathogenic	
RFXANK	AR	1	*NM_003721.4:c.232C>T (p.Arg78*) STOP_GAINED*	Pathogenic	This variant is not present in population databases (ExAC no frequency).
CD40LG	X	1	*NM_000074.3: SPLITE_SITE_ACCEPTOR*	Pathogenic	
**COMBINED IMMUNODEFICIENCIES WITH ASSOCIATED OR SYNDROMIC FEATURES**					
22q11.2DS	AD	12	*22q11.2 del 22*	Pathogenic	22q11.2DS
NBN	AR	5	*NM_002485.5:c.657_661del (p.Lys219fs)*	Pathogenic	
STAT3	AD	1	*NM_139276.3:c.1144C>T (p.Arg382Trp)*	Pathogenic	
		1	*NM_139276.3:c.1850G>A (p.Gly617Glu)*	Likely pathogenic	

		1	*NM_139276.3:c.1910T>C (p.Val637Ala)*	Pathogenic	
STAT5b	AR	1	*NM_012448.4: c.452T>C (p.L151P)*	Pathogenic	
**PREDOMINANTLY ANTIBODY DEFICIENCIES**					
BTK	X-linked	1	*NM_000061.3:del exones 2-19 whole gene gone*	Pathogenic	
		2	*NM_000061.3:c.557dupA p.(Pro187AlafsTer7)*	Pathogenic	Siblings
		2	*NM_000061.3:c.17605>A p.(Met587Lys)*	Pathogenic	Siblings
CARD11	AD	1	*NM_000061.3:c.2368G>A (p.Ala790Thr)*<br>*NM_000061.3:c.2917C>T (p.Arg973Cys)*	Likely pathogenic	Compound heterozygosity
TNFRSF13 B	AR	1	*NM_012452.3:c.204dupA (p.Leu69ThrfsTer12)*	Likely pathogenic	
		1	*NM_012452.3:c.311G>A (p.Cys 104Tyr)*	Pathogenic	
		1	*NM_012452.3:c.431C>G (p.Ser144*)*	Pathogenic	
		1	*NM_012452.3:c.310T>C (p.Cys104Arg)*	Likely pathogenic	
IL12RB1	AR	1	*NM_005535.3:c.942C>A (p.Tyr314*)*	Pathogenic	The same patient has c.*310T>C (p.Cys104Arg)* in *TNFRSF13B* (listed above)
**DISEASES OF IMMUNE DYSREGULATION**					
FASLG	AR	1	*NM_000639.1:c.259T>C*	Likely pathogenic	

SH2D1A	X-linked	1	*NM_002351.5:Deletion (Entire coding sequence)*	Pathogenic	
CTLA4	AD	1	*Deletion (Entire coding sequence)*	Pathogenic	
**CONGENITAL DEFECTS OF PHAGOCYTE NUMBER, FUNCTION, OR BOTH**					
MPO	AR	1	*NM_000250: c.2031-2A>C*	Pathogenic	
CYBB	X-linked	1<br>1	*NM_000397:c.252+5G>C*<br>*NM_000397.3:c.1165G>A*	Likely pathogenic<br>Likely pathogenic	Complete clinical correlation<br>Female carrier with mild clinical symptoms
LAD1	AR	1	*NM_005558.4:***c.***616C>T(p.His206Tyr)*	Pathogenic	
ELANE	AD	1	*NM_001972.3: g.1287A>G start_lost*	Pathogenic	This variant is not present in population databases (ExAC no frequency).
ITGB2	AR	1	*NM_000211.5;***c.***616C>T (p.His206Tyr)*	Pathogenic	
NCF1	AR	1<br>1	*NM_000265.5:c.269G>A (p.Arg90His)*<br>*NM_000265.5:c.75_76delGT(p.Tyr26HisfsTer 26)*	Likely Pathogenic<br>Pathogenic	
**AUTO-INFLAMMATORY DISORDERS**					
MEFV	AD	1	*NM_000243.2:c.2080A>G(p.Met694Val)*	Pathogenic	
		1	*NM_000243.2:c.2230G>T (p.Ala744Ser)*	Pathogenic	
		1<br>1	*NM_000243.2:c2177T>C (p.Val726Ala)*<br>*NM_000243.2:c.2040G>C (p.Met680Ile)*	Pathogenic<br>Pathogenic	
MVK	AR	1	*NM_000431.3:c.1006G>A(p.Gly336Ser)*	Likely Pathogenic	

		1	*NM_000431.3:c.1129G>A(p.Val377Iie)*	Pathogenic	
		1	*NM_000431.3:c.803T>C p.(Ile268Thr);*<br>*NM_000431.3:c.1129G>A p.(Val377Ile)*	Pathogenic	Compound heterozygosity
STING1	AD	1	*NM_198282.4:c.461A>G(p.Asn154Ser)*	Pathogenic	
**COMPLEMENT DEFICIENCIES**					
MASP2	AD	1	*NM_006610.4:* ***c.359A>G(p.Asp120Gly)***	Likely pathogenic	
